# *XAF1*基因对肺腺癌细胞A549的作用及机制

**DOI:** 10.3779/j.issn.1009-3419.2014.12.01

**Published:** 2014-12-20

**Authors:** 东来 陈, 福全 张, 永华 桑, 蓉英 朱, 洪涛 张, 勇兵 陈

**Affiliations:** 1 215123 苏州，苏州大学独墅湖校区2012级临床医学3班 The Dushu Lake Campus Class 3 Grade, 2012 Clinical Medicine, Soochow University, Suzhou 215123, China; 2 215004 苏州，苏州大学附属第二医院 The Second Hospital Affiliated to Suzhou University, Suzhou 215123, China; 3 215123 苏州，苏州大学癌症分子遗传学实验室 Cancer Molecular Genetics Laboratory of Soochow University, Suzhou 215123, China

**Keywords:** *XAF1*基因, 肺肿瘤, 增殖, 凋亡, X-linked inhibitor of apoptosis protein, Lung adenocarcinoma, Proliferation, Apoptosis

## Abstract

**背景与目的:**

XAF1是重要的肿瘤细胞生长抑制因子，其低表达与多种肿瘤细胞有关。研究肿瘤抑制基因*XAF1*对人肺腺癌细胞株A549的作用及机制。

**方法:**

利用重组腺病毒Ad5/F35-XAF1和对照腺病毒Ad5/F35-NULL瞬时转染A549细胞，用逆转录聚合酶链式反应（reverse transcriptase polymerase chain reaction, RT-PCR）和Western blot方法检测A549细胞株中XAF1 mRNA和蛋白质的表达；MTT检测细胞增殖率、流式细胞仪检测细胞凋亡率，并用Western blot法检测凋亡相关蛋白的表达。

**结果:**

腺病毒介导的XAF1瞬时转染肺腺癌A549细胞后，XAF1 mRNA及蛋白表达水平明显提高，并能明显抑制该细胞增殖和促进细胞凋亡，蛋白质印记法显示凋亡相关蛋白PARP、Caspase-3、Caspase-8的裂解条带。

**结论:**

恢复*XAF1*基因在人肺腺癌A549细胞中表达后，能明显抑制该肿瘤细胞增殖并促进其凋亡，其机制可能与XAF1激活肺癌细胞相关凋亡途径有关。

## Abstract (en)

**Background and objective:**

XAF1 is a factor necessary to inhibit tumor cell growth. Low XAF1 expression is associated with various tumor cells. The aim of this study is to investigate the effect and the mechanism of adenovirus vector Ad5/F35 mediated X-linked inhibitor of apoptosis protein associated factor-1 (XAF1) on the inhibition of cell proliferation and the induction of apoptosis of human lung adenocarcinoma cell A549.

**Methods:**

Recombinant virus Ad5/F35-XAF1 and controlled virus Ad5/F35-NULL exhibited different multiplicities of infection (MOI) at the same time. mRNA and protein expressions of XAF1 were determined by reverse transcriptase polymerase chain reaction (RT-PCR) and Western blot, respectively. Cell proliferation was observed by methyl thiazolyl tetrazolium (MTT) assay, and cell apoptosis was analyzed by FACS with Annexin V-FITC/PI double staining. The expressions of apoptosis-associated proteins, such as PARP, Caspase-3, and Caspase-8, were also determined by Western blot.

**Results:**

mRNA and protein expressions of XAF1 were significantly increased in human lung adenocarcinoma cell A549 after this cell was transfected with Ad5/F35-XAF1 for 48 h; these expressions were higher than those of the controlled group Ad5/F35-NULL. Cell proliferation was inhibited and apoptosis was induced in a dose-dependent manner in the Ad5/F35-XAF1 group. After Ad5/F35-XAF1 transfection was performed, the cleavage of apoptosis-associated proteins, such as PARP, Caspase-3, and Caspase-8, was activated.

**Conclusion:**

Restored XAF1 expression inhibits cell proliferation and induces cell apoptosis in human lung adenocarcinoma cell line A549. Furthermore, XAF1 may activate associated apoptotic signaling pathways in A549 cell line.

细胞的凋亡抑制在肺癌的发生发展中起重要作用^[1]^。X连锁凋亡抑制蛋白（X-linked inhibitor of apoptosis, XIAP）相关因子1（*XAF1*）是新鉴定的肿瘤抑制基因，在许多肿瘤组织和肿瘤细胞株中低表达甚至不表达^[2]^。新近研究^[3, 4]^发现，XAF1在肿瘤细胞中高表达可抑制胃癌细胞、肺鳞癌细胞的生长，并诱导肿瘤细胞凋亡，增加肿瘤细胞对抗肿瘤药物的敏感性。但对肺腺癌细胞作用如何尚未见相关研究报道。本实验以重组腺病毒为载体，研究过表达*XAF1*基因对人肺腺癌细胞A549的增殖和诱导细胞凋亡的作用及机制，为腺病毒介导*XAF1*基因治疗肺腺癌提供实验依据。

## 1 材料与方法

### 1.1 材料

逆转录试剂盒购自Promega公司；甲基噻唑基四唑（MTT）、二甲基亚砜（DMSO）购自Sigma公司，Annexin V-FITC/PI凋亡检测试剂盒购自BD公司；RIPA裂解液购自上海申能博彩公司；XAF1抗体购自Abcam公司；PARP、Caspase-3和Caspase-8抗体购自Cell Signaling Technology公司；β-actin抗体购自Sigma公司；RNA提取试剂盒和辣根过氧化物酶连接的二抗购自北京康为世纪公司；增强化学发光荧光试剂ECL购自Amersham bioscience公司；XAF1和β-actin引物由上海生工生物公司合成，以β-actin为内参照。

### 1.2 细胞培养

人肺腺癌A549细胞购自中国科学院上海细胞生物学研究所，为贴壁细胞，用含10%胎牛血清（FBS）的RPMI-1640培养基（Gibco公司）培养和传代（37 ℃、5%CO_2_）。

### 1.3 重组腺病毒

Ad5/F35-XAF1和对照空病毒Ad5/F35-Null由北京本元正阳公司合成与扩增，转染滴度分别0.69×10^10^ PFU/mL和1.26×10^10^ PFU/mL。

### 1.4 逆转录聚合酶链式反应（reverse transcriptase polymerase chain reaction, RT-PCR）

采用试剂盒抽取细胞总RNA，测定RNA浓度，并逆转成cDNA。XAF1引物上游：5'-TCCGCAATTCATGCTCCACGAGTCCTA CTG-3'，下游：5'-ACGCGTCGACAAACTCTGAGTCTGGACAAC-3'，产物大小260 bp。内参照β-actin引物上游：5'-ATCTGGCACCACACCTTCTACAATGAGCTGC-3'，下游：5'-CGTCATACTCCTGCTTGCTGATCCACATCTGC-3'，产物大小830 bp。PCR反应条件：95 ℃、3 min；94 ℃、45 s，57 ℃、45 s，72 ℃、45 s，共30个循环。最后72 ℃延伸6 min。PCR产物行2%琼脂糖凝胶电泳，100伏电压30 min电泳后在凝胶成像系统上拍照和分析。

### 1.5 MTT法检测细胞生长活力

将对数生长期的A549原代肺腺癌细胞以5, 000个/孔接种在96孔板上，24 h后，用无血清RPMI-1640稀释重组腺病毒Ad5/F35-NULL和Ad5/F35-XAF1，分别按MOI（50、100、200）加入96孔板中转染细胞，空白对照组不加任何病毒处理，置于37 ℃、5%CO_2_温箱孵育4 h后置换为含10%FBS的RPMI-1640完全培养液，继续培养48 h后收集细胞，弃去上清，每孔加入5 mg/mL的MTT 20 μL，在培养箱孵育4 h，小心吸净MTT液，后加入二甲基亚砜（DMSO）150 mL/孔，避光摇床上放置10 min，于波长570 nm处测定吸光度（*A*）值，细胞增殖率=（实验孔*A*值-空白对照孔*A*值）/对照孔*A*值×100%。各滴度梯度组均设3个复孔，每组实验重复3次，取平均值。

### 1.6 Annexin V-FITC/PI双染法（流式细胞仪法）

将A549原代肺腺癌细胞以2×10^5^/孔密度接种于6孔板中，按MOI 100的浓度分别加入Ad5/F35-NULL和Ad5/F35-XAF1（方法同5）。转染4 h后加入含10%FBS的RPMI-1640完全培养液，继续37 ℃温箱孵育48 h后制成细胞悬液。加入Annexin V-FITC和PI染色液，上流式细胞仪检测细胞凋亡率，每组重复3次，取平均值。

### 1.7 Western blot检测XAF1及凋亡相关蛋白的表达

用RIPA细胞裂解液抽取细胞总蛋白，并用BCA方法测蛋白浓度，取50 μg蛋白质，加1/4蛋白量的5×蛋白上样缓冲液，配置10%十二烷基磺酸钠-聚丙烯酰胺凝胶（SDS-PAGE）并上样，依次进行蛋白垂直电泳、转膜、5%脱脂牛奶封闭，分别加入特异性β-actin抗体（1:5, 000）和XAF1抗体（1:1, 000）以及凋亡相关蛋白Caspase-3抗体（1:1, 000）、Caspase-8（1:1, 000）、PARP抗体（1:1, 000），4 ℃孵育过夜，用TBS/T洗膜后分别加入对应二抗，室温摇床2 h，ECL显像，在凝胶成像系统上拍照并分析。

### 1.8 统计学方法

应用SPSS 16.0统计软件，组间比较采用单因素方差分析（*ANOVA*）法，*P* < 0.05为差异有统计学意义。

## 2 结果

### 2.1 人肺腺癌细胞株A549中XAF1 mRNA和蛋白的表达

Ad5/F35-XAF1瞬时转染A549细胞48 h后，行PCR和Western blot法检测细胞中mRNA和蛋白的表达。结果显示，与Ad5/F35-NULL转染组相比，A549细胞中XAF1 mRNA和蛋白表达在Ad5/F35-XAF1转染48 h后明显增高（图 1）。

**1:**
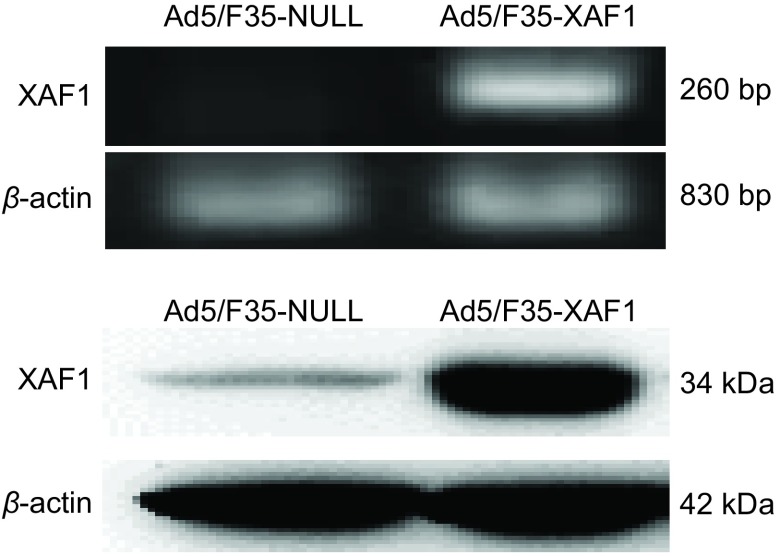
重组腺病毒感染后A549细胞中XAF1 mRNA（A）和蛋白（B）的表达明显增强 After infecting by recombinant adenovirus, the expression of mRNA (A) and protein (B) of *XAF1* gene increased significantly in A549 cell line

### 2.2 细胞增殖率情况

将A549原代细胞、转染Ad5/F35-XAF1和Ad5/F35-NULL三组细胞培养48 h后，MTT法提示肺腺癌A549细胞增殖率随着Ad5/F35-XAF1 MOI的升高而降低，当MOI为100和200时，细胞增殖抑制率分别为19%和52%，两者比较有统计学差异；而瞬时转染Ad5/F35-NULL 48 h后，各MOI组细胞增殖率无明显统计学差异，并且MOI为100和200时，细胞增殖抑制率分别为3%和10.5%，Ad5/F35-XAF1组细胞增殖抑制率明显低于相应Ad5/F35-NULL组（*P* < 0.05），其中转染Ad5/F35-XAF1和Ad5/F35-NULL两组A549细胞增殖抑制率测算均以A549原代细胞为标准（图 2）。

**2:**
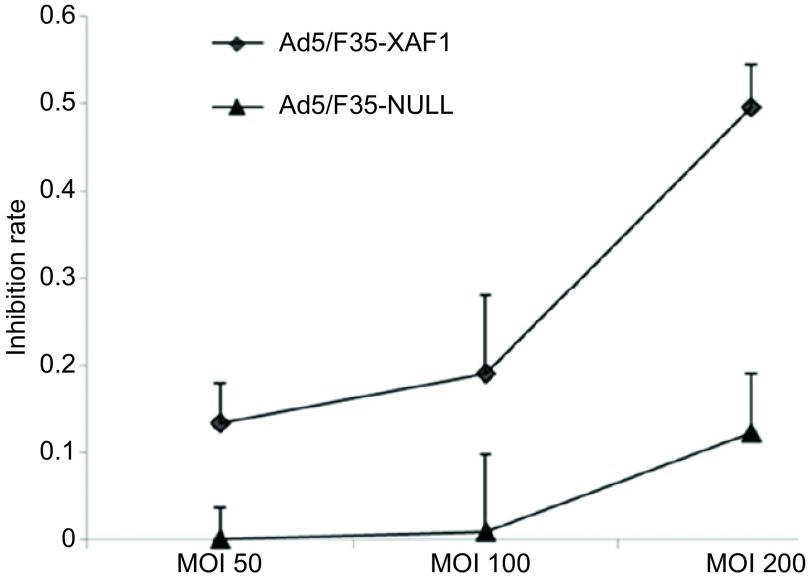
XAF1高表达明显抑制了A549细胞的增殖 The higer expression of *XAF1* gene inhibited A549 cell proliferation significantly

### 2.3 细胞凋亡率检测情况

将A549原代细胞、转染Ad5/F35-XAF1和Ad5/F35-NULL三组细胞培养48 h后，Annexin V-FITC/PI双染法于流式细胞仪上机检测。结果显示，A549原代细胞、Ad5/F35-NULL及Ad5/F35-XAF1三组中A549细胞早期凋亡率分别为3.2%、6%和15%。与Ad5/F35-NULL及A549原代细胞两组相比，Ad5/F35-XAF1转染组中A549细胞早期凋亡率明显增高，差异有统计学意义（*P* < 0.05）（图 3）。

**3:**
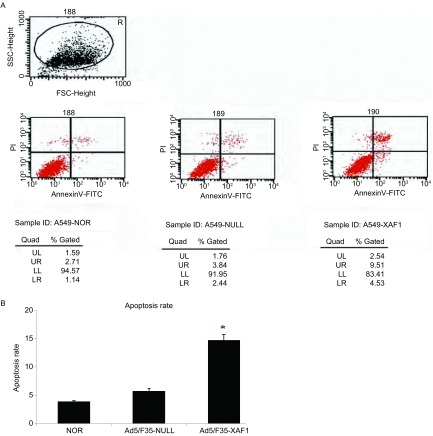
Annexin V-FITC/PI双染法检测A549细胞凋亡率。A：原始图；B：统计柱状图。Ad5/F35-XAFl转染组中A549细胞早期凋亡率明显增高。 Apoptosis rate of A549 detected with Annexin V-FITC/PI staining method. A: Raw data; B: Statistical histogram. Early stage apoptosis rate of A549 cell line increased significantly in the Ad5/F35-XAF1 transfected group.

### 2.4 凋亡相关蛋白的表达

Ad5/F35-XAF1和Ad5/F35-NULL分别转染肺腺癌A549细胞48 h后，用Western blot法检测凋亡相关蛋白的表达。与对照病毒相比，Ad5/F35-XAF1组的A549细胞中PARP、Caspase-3和Caspase-8蛋白出现明显裂解条带，显示Caspase依赖途径的凋亡通路活化（图 4）。

**4:**
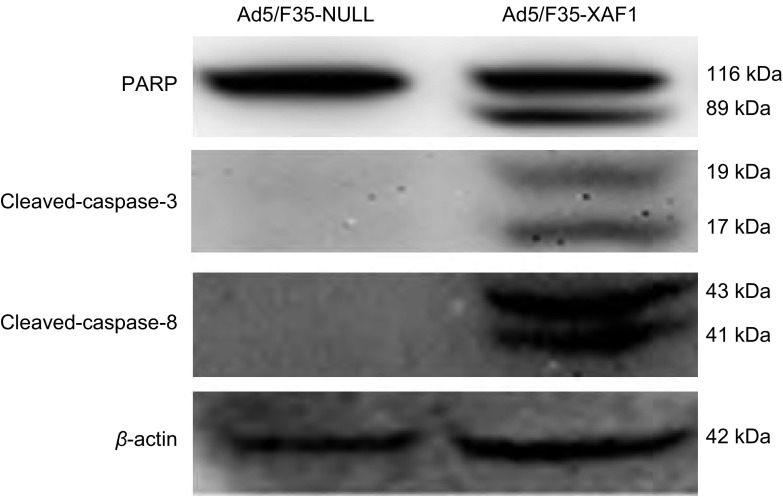
肺癌A549细胞凋亡相关蛋白的表达（蛋白质印记法） The expression of apoptosis related proteins in human lung cancer cell line A549

## 3 讨论

XAF1是运用酵母杂交法发现的一种新型XIAP拮抗蛋白，可直接与XIAP结合并抑制其抗凋亡作用^[5, 6]^，并发现XAF1对凋亡抑制家族IAPs家族有普遍的抑制作用^[7]^。本课题组已有的研究^[4]^表明，XAF1在人肺鳞癌组织中的表达明显低于正常肺组织，且XAF1低表达的程度与肺鳞癌患者的肿瘤分期、病理分级、肿瘤浸润、淋巴结转移等密切。其他研究也发现，通过腺病毒介导的XAF1能恢复*XAF1*基因在胃癌^[3]^、结肠癌^[8]^等消化系统肿瘤细胞中表达甚至过表达，且能抑制细胞增殖促进细胞凋亡。

作为重要的肿瘤抑制因子之一，XAF1在人食管癌^[9]^、肺癌^[4]^、胃肠癌^[10]^等多种肿瘤组织中的表达明显低于正常组织，且与肿瘤的分期、分级相关，但其在肺腺癌A549中抑制肿瘤细胞生长的机制尚不明确。本研究以重组腺病毒为载体，恢复*XAF1*基因在肺腺癌A549细胞中的表达，用PCR及Western blot方法均提示XAF1表达明显增加，而对照病毒Ad5/F35-NULL组中XAF1表达低下（图 1）。用MTT法检测Ad5/F35-XAF1对A549细胞活力的影响，结果提示Ad5/F35-XAF1组中其细胞活力较相同MOI值的对照空病毒Ad5/F35-NULL明显降低（图 2）。流式细胞学结果显示，恢复XAF1在A549细胞中的表达后可明显诱导该肿瘤细胞凋亡（图 3）并且凋亡相关蛋白Caspase-3、Caspase-8和PARP的裂解条带明显增加（图 4），表明*XAF1*基因诱导A549细胞凋亡的途径主要通过Caspase依赖的凋亡信号通路。Liston等^[11]^研究发现，XAF1与XIAP结合后定位于细胞核，它通过竞争性抑制XIAP对Caspase-3抑制作用以激活Caspase-3、Caspase-7、Caspase-9的活性，从而诱导肿瘤细胞的凋亡，我们的研究证实了他的部分研究结果。

综上所述，恢复*XAF1*基因在肺腺癌A549细胞中的表达，可明显抑制该肿瘤细胞的增殖，并可明显诱导其凋亡，其机制可能与其激活Caspase凋亡信号通路有关，*XAF1*基因有望成为治疗肺癌的新靶点。
